# Immunosuppression overcomes insulin- and vector-specific immune responses that limit efficacy of AAV2/8-mediated insulin gene therapy in NOD mice

**DOI:** 10.1038/s41434-018-0052-5

**Published:** 2018-12-04

**Authors:** Asha Recino, Shu Uin Gan, Kian Chuan Sia, Yvonne Sawyer, Jenny Trendell, Richard Kay, Fiona M. Gribble, Frank Reimann, Rob Foale, Maria Notaridou, Nick Holmes, Andrew Lever, Kok Onn Lee, Amit Nathwani, Anne Cooke, Roy Calne, Maja Wallberg

**Affiliations:** 1 Department of Pathology, University of Cambridge, Tennis Court Road, Cambridge, CB2 1QP UK; 2 Department of Surgery, National University of Singapore, Singapore, Singapore; 3 Institute of Metabolic Science, Addenbrooke’s Hospital, Cambridge, UK; 4 Dick White Referrals, Station Farm, Six Mile Bottom, Suffolk, UK; 5 Department of Haematology, UCL Cancer Institute, London, UK; 6 Department of Medicine, University of Cambridge, Cambridge, UK; 7 Department of Medicine, National University of Singapore, Singapore, Singapore; 8 Department of Surgery, University of Cambridge, Cambridge, UK

**Keywords:** Immunological disorders, Autoimmunity, Adaptive immunity

## Abstract

We report the restoration of euglycaemia in chemically induced diabetic C57BL/6 mice and spontaneously diabetic Non Obese Diabetic (NOD) mice by intravenous systemic administration of a single-stranded adeno-associated virus (ssAAV2/8) codon optimised (co) vector encoding furin cleavable human proinsulin under a liver-specific promoter. There were no immunological barriers to efficacy of insulin gene therapy in chemically induced C57BL/6 mice, which enjoyed long-lasting correction of hyperglycaemia after therapy, up to 250 days. Euglycaemia was also restored in spontaneously diabetic NOD mice, although these mice required a 7–10-fold higher dose of vector to achieve similar efficacy as the C57BL/6 mice and the immunodeficient NOD^*scid*^ mice. We detected CD8^+^ T cell reactivity to insulin and mild inflammatory infiltration in the livers of gene therapy recipient NOD mice, neither of which were observed in the treated C57BL/6 mice. Efficacy of the gene therapy in NOD mice was partially improved by targeting the immune system with anti-CD4 antibody treatment, while transfer of NOD mouse AAV2/8-reactive serum to recipients prevented successful restoration of euglycaemia in AAV2/8-HLP-hINSco-treated NOD^*scid*^ mice. Our data indicate that both immune cells and antibodies form a barrier to successful restoration of euglycaemia in autoimmune diabetic recipient mice with insulin gene therapy, but that this barrier can be overcome by increasing the dose of vector and by suppressing immune responses.

## Introduction

The beta cells of the pancreas produce insulin, which in turn induces upregulation of the glucose transporter GLUT4 in many cell types, allowing efficient glucose uptake. When the beta cells are destroyed or incapacitated, as happens in type 1 diabetes, insulin is no longer produced in sufficient amounts and the tissues are unable to metabolise glucose despite it being abundant in the circulation. This disease was deadly until the 1920s when the insulin protein was identified [1], and administration of exogenous insulin has remained the available therapy for the disease since this discovery.

The success of gene therapy introducing hepatic expression of clotting factors in people with haemophilia B [2–4], and more recently expanding to other disorders [5, 6], demonstrates that this approach is feasible in human patients. The possibility of producing insulin endogenously via gene therapy is appealing as a form of basal long-acting insulin. We have demonstrated that the vector used for haemophilia therapy can be successfully modified to express insulin, and can restore euglycaemia in immunocompromised chemically induced diabetic mice [7]. Other groups have previously achieved restoration of insulin production through gene therapy using either naked DNA [8, 9] or various viral vectors [10–16] in animal models, as reviewed in [17]. These studies show that the insulin gene can be expressed in vivo after gene therapy, and can restore euglycaemia in diabetic hosts that have been rendered diabetic by chemical ablation of the beta cell population. However, type 1 diabetes is very different from haemophilia, and even if insulin can be expressed successfully there are additional challenges that need to be overcome before insulin gene therapy could be considered in people with type 1 diabetes. One of those challenges is the autoimmune aetiology of the disease, with autoreactive T cells reported as the cause of beta cell destruction (reviewed in [18]). The recurrence of autoimmunity, resulting in the destruction of the de novo insulin expressing tissue, is an obvious concern.

We were interested in whether hepatic expression of insulin mediated via the previously described AAV2/8-based vector [7] could restore euglycaemia even in a completely immunocompetent host, particularly where there is already pre-existing autoimmunity to the insulin protein. In people with haemophilia, the occurrence of T cells reactive against the transgenic blood clotting factor IX coincided with reduced expression of the transgene, and treatment with glucocorticoids was required to stabilise the transgene expression [2]. As insulin-producing beta cells are a major target for the autoimmune response responsible for destroying the beta cells in the first place [19, 20], it is not unreasonable to expect that even if transgenic insulin can be expressed, the autoreactive T cells and B cells may become reactivated to destroy the successfully transfected cells. Studies of pancreas transplantation have demonstrated that even when a diabetic islet graft recipient received a completely human leucocyte antigen-matched graft from an identical twin, reactivation of anti-islet immune responses led to rapid destruction of the graft [21]. In diabetic NOD mice, syngeneic islet grafts are destroyed within 10 days unless potent immunosuppression is administered [22, 23]. Immune responses to the vector itself can also limit the efficacy of the treatment [24]. Thus, we were concerned that long-term restoration of insulin production in mice with autoimmune diabetes would be compromised by recurring immune responses destroying the insulin-producing hepatocytes.

## Results

### Spontaneously diabetic NOD mice require higher doses of AAV2/8-HLP-hINSco-mediated gene therapy to restore euglycaemia

**Fig. 1:**
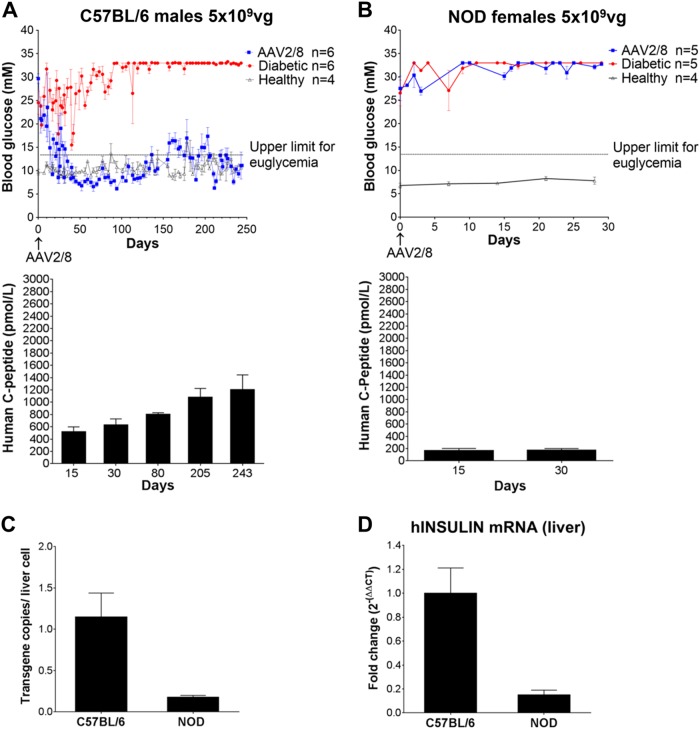
Administration of 5 × 10^9^ vg/mouse AAV2/8-HLP-hINSco restores normoglycaemia in chemically induced diabetic C57BL/6 mice but not in naturally diabetic NOD mice. C57BL/6 male mice (**a**), rendered diabetic with streptozotocin (40 mg/kg i.p. for 5 days), or NOD female mice (**b**), tested positive for diabetes more than three consecutive times, were treated with AAV2/8-insulin therapy (indicated as AAV2/8) (day 0). Blood glucose levels were then monitored over time (**a,** **b** top panels). Plasma human C-peptide levels of C57BL/6 (**a**, bottom panel) and NOD (**b**, bottom panel) mice were measured over time after AAV2/8-HLP-hINSco injection. **c** Average genome copy number of virus per liver cell at the end of the 30-day experiments. **d** Transgenic human insulin mRNA expression in the treated C57BL/6 and NOD mice livers relative to C57BL/6 mice’s levels at the end of the 30-day experiments. Data shown are expressed as mean ± SE and are representative of two independent experiments

**Fig. 2:**
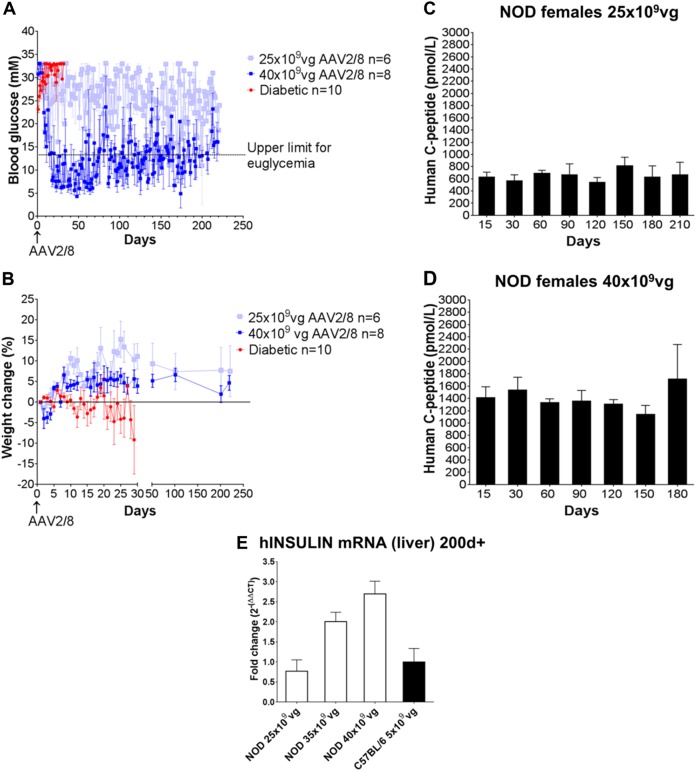
Increased dose of the vector allows to better control blood glucose levels in diabetic NOD mice. **a** Established diabetic NOD females were injected with either 25 × 10^9^ vg/mouse (**a**, light blue) or 40 × 10^9^ vg/mouse (**a**, dark blue) AAV2/8-HLP-hINSco vector (indicated as AAV2/8), or vehicle only (**a**, red line) (day 0) and were then monitored for blood glucose levels. **b** Percentage of weight change from baseline for the mouse groups in **a**. **c**, **d** Plasma human C-peptide levels measured over time after AAV2/8-HLP-hINSco injection. **e** Transgenic human insulin mRNA expression in the livers of NOD and C57BL/6 mice transduced with different doses of AAV2/8-HLP-hINSco as indicated in the figure. Expression is relative to C57BL/6 levels and was determined after more than 200 days from infection. Data shown are expressed as mean ± SE and are representative of two independent experiments

Male C57BL/6 mice were injected with streptozotocin to destroy the beta cells, and female NOD mice were monitored for diabetes and selected for treatment once consecutive tests proved them to be irreversibly diabetic. They were then injected with AAV2/8-HLP-hINSco at a dose of 5 × 10^9^ viral genomes (vg)/mouse, which had previously proven successful in immunocompromised mice [7], and which we confirmed in our colony of NOD^*scid*^ mice (S1). This dose completely restored euglycaemia in diabetic C57BL/6 mice (Fig. 1a), indicating that the presence of a fully functional immune system did not affect transduction and expression of the transgene. However, the same dose had no effect on glycaemia in spontaneously diabetic NOD mice (Fig. 1b), although the treated mice maintained stable body weight (S2). When tested for transgenic human C-peptide, a cleaved product of proinsulin which is used as read-out of insulin production, diabetic NOD mice had lower levels in serum after treatment (Fig. 1b, bottom panel) than C57BL/6 (Fig. 1a, bottom panel), indicating less effective transduction of the vector (or survival of the transduced cells). This result was further supported by the lower average of genome copy number of virus per liver cell (Fig. 1c) and the lower expression of human insulin mRNA in the liver (Fig. 1d) in NOD mice. When the dose was increased five-fold, to 25 × 10^9^ (Fig. 2a), we observed a lowering of blood glucose in the naturally diabetic female NOD mice. An additional increase to 35 × 10^9^ vg did not allow complete control of hyperglycaemia either (data not included). Only when the dose was further increased to 40 × 10^9^ vg the diabetic NOD mice were restored to normal blood glucose levels (Fig. 2a). A dose of 50 × 10^9^ was also administered, but the mice had to be culled due to sustained severe hypoglycaemia (data not included). Even though the lower dose of 25 × 10^9^ did not restore normoglycaemia in the diabetic mice, the insulin produced was enough to maintain a normal body weight gain (Fig. 2b). Increasing the dose correlated with increased levels of transgenic human C-peptide in the serum of the treated mice (Fig. 2c, d) and increased expression of transgenic human insulin mRNA in the liver (Fig. 2e).

The transgenic insulin is secreted continuously and is not regulated by blood glucose levels and incretin hormones. This can cause hypoglycaemia unless sufficient amounts of glucose are ingested, which we observed when titrating the vector dose in diabetic NOD mice. We therefore investigated whether withdrawal of food would cause hypoglycaemia episodes in the mice receiving tolerated doses of AAV2/8-HLP-hINSco. We observed a lowering of blood glucose levels in all NOD mice, including the untreated diabetic ones, after overnight fasting, but none dipped to levels below 3.3 mM (S3).

The difference in effect was not due to the sex difference [25] between the female NOD mice and the male C57BL/6, as chemically induced diabetic male NOD mice also resisted restoration of euglycaemia with 5 × 10^9^ vg/mouse (S4A), and had human C-peptide levels similar to the spontaneously diabetic female NOD mice treated with the same dose (S4B). Male NOD mice have activation of islet-specific immune responses and develop inflammatory infiltrates in the pancreatic islets just like the female mice, but progress to diabetes less frequently. This necessitates the use of streptozotocin or other drugs to precipitate disease in the males, but the mice also have the underlying anti-islet immune reactivity and fully functional NOD mouse immune system.

Diabetic NOD mice treated with 25 × 10^9^ vg achieved similar levels of serum human C-peptide as the C57BL/6 mice treated with 5 × 10^9^ vg (a five-fold increase of the dose) (Fig. 2c vs Fig. 1a). An even higher dose was necessary to achieve adequate glucose control (Fig. 2a). If diabetic NOD were insulin resistant, this could explain the requirement for more insulin to restore euglycaemia. An insulin tolerance test was therefore performed, where the rapidity and magnitude of the blood glucose-lowering effect of a bolus injection of insulin was recorded (S5A). This demonstrated no difference in the response in NOD mice (S5B) and C57BL/6 (S5C). If anything, the diabetic C57BL/6 mice showed a less striking response to insulin challenge than the diabetic NOD mice. In addition, healthy NOD mice and C57BL/6 mice secreted comparable amounts of insulin in response to glucose challenge (S5D), indicating that NOD mice do not normally require more insulin to control blood glucose.

### Transgenic insulin in AAV2/8-HLP-hINSco-transfected liver cells reactivates cellular immune responses in NOD mice

**Fig. 3:**
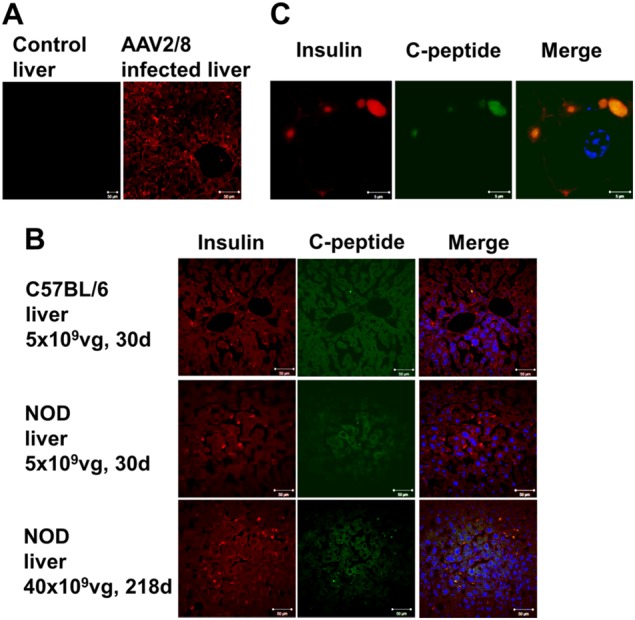
Transgenic insulin is stored in intracellular granules of AAV2/8-insulin-transduced liver cells. Healthy control (**a**, left) and AAV2/8-HLP-hINSco-treated (indicated as AAV2/8) (**a**, right) NOD mouse liver sections stained for insulin. Co-localisation of insulin (red) and C-peptide (green) in liver sections of C57BL/6 mice (**b**, top panel) and NOD female mice (**b**, middle panel) injected with 5 × 10^9^ vg AAV2/8-HLP-hINSco after 30 days, and NOD female mice injected with 40 × 10^9^ vg after 218 days (**b**, bottom panel). Scale bar 50 μm. Magnification of a transduced liver cell in **c**, where scale bar is 5 μm. Blue shows nuclear staining. Images are representative of at least two experiments

**Fig. 4:**
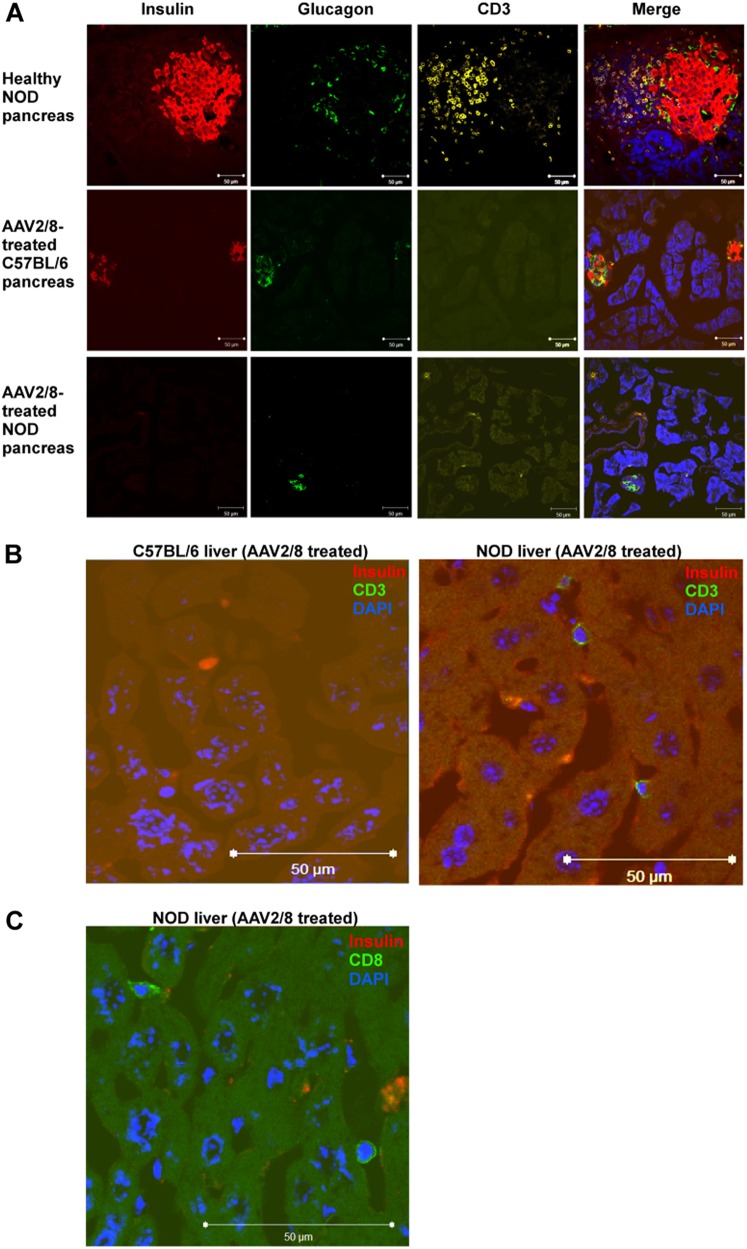

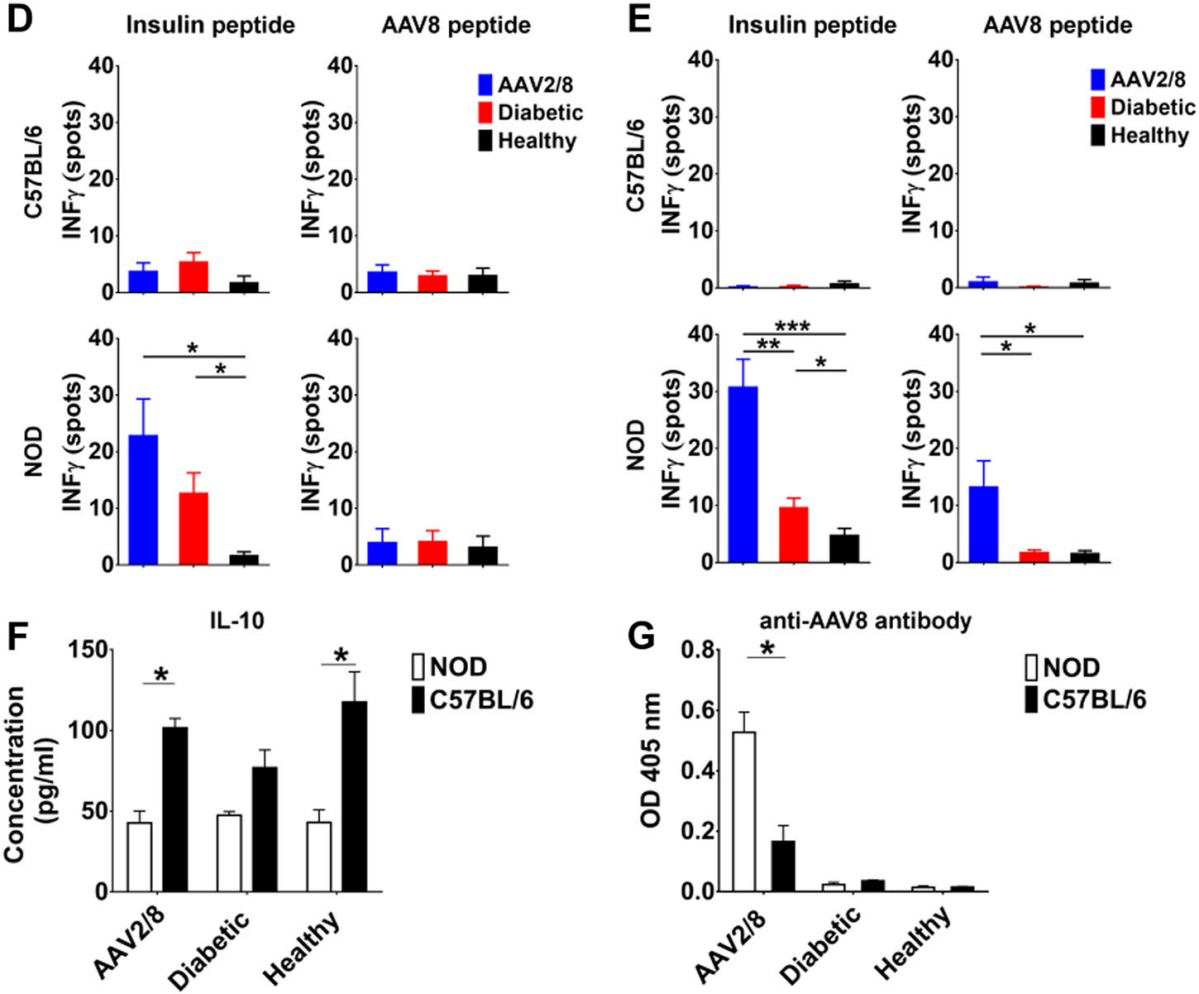
AAV2/8-insulin-based therapy elicits a cellular immune response against the vector and the transgene in NOD diabetic mice. Livers of NOD mice treated with AAV2/8-HLP-hINSco (indicated with AAV2/8) show signs of T cell infiltration observed in pre-diabetic NOD mouse pancreata. **a** Pancreas sections from a 8-week-old control NOD female pancreas (**a**, top panel), a 30-day AAV2/8-insulin-treated C57BL/6 (**a**, middle panel) and a 30-day AAV2/8-insulin-treated NOD (**a**, bottom panel) were stained for insulin (red) and glucagon (green), and CD3 (yellow) to show β-cell destruction in treated mice. **b** Livers from AAV2/8-HLP-hINSco -injected C57BL/6 (**b**, left) and NOD (**b**, right) mice were stained for insulin and CD3 to test for T cell infiltration. **c** Livers from AAV2/8-HLP-hINSco-injected NOD mice were stained for insulin and CD8. Blue shows nuclear DAPI staining. Scale bar 50 μm. **d, e** Ex vivo stimulated splenocytes from AAV2/8-HLP-hINSco -treated NOD mice and C57BL/6 controls for IFN-γ ELISPOT assay. Cells from NOD and C57BL/6 mice were harvested after 30 days (**d**) or more than 200 days (**e**) from the day of the injection of the AAV2/8-insulin vector. Cells were stimulated in vitro for 40 h with CD8^+^ immunodominant peptides (either InsB_15–23_ peptide for NOD or insulin K^b^-restricted epitope A_12–21_ containing peptide for C57BL/6) or the AAV8 capsid-specific CD8^+^ T cell peptide NSLANPGIA and the number of resulting IFN-γ spots was counted with an ELISPOT reader. **f** Levels of anti-inflammatory cytokines in NOD mice compared to C57BL/6. Splenocytes from C57BL/6 and NOD mice were harvested at the end of a 30-day treatment with AAV2/8-HLP-hINSco 5 × 10^9^ vg and stimulated with PMA/Iono for 48 h. Supernatants were then collected and tested for IL-10 production. **g** Mouse anti-AAV8 ELISA performed on blood plasma of diabetic, healthy and 5 × 10^9^ vg AAV2/8-HLP-hINSco-treated NOD and C57BL/6 mice. Treated mice were tested for anti-AAV8 antibodies 30 days after the beginning of the therapy. The absorbances at 405 nm correlate with the concentration of the antibody. *(*p* ≤ 0.05), **(*p* ≤ 0.01), ***(*p* ≤ 0.001), unpaired Student’s *t*-test. Data shown are expressed as mean ± SE and are representative of two independent experiments

Both insulin (Fig. 3a, b) and C-peptide (Fig. 3b) can be detected in the livers of treated C57BL/6 mice (Fig. 3b, top) and NOD mice (Fig. 3b, middle and bottom panels), but not in untreated controls (Fig. 3a, left). Closer examination reveals colocalization of insulin and C-peptide staining on a cellular level (Fig. 3c). However, the initial destruction of the pancreatic beta cells in the NOD mice is immune mediated (Fig. 4a, top panels), while the chemically induced diabetic C57BL/6 mice lack immune infiltration around the sparse insulin-positive cells that remain after treatment (Fig. 4a, middle panels). Analysis of total endogenous insulin mRNA corroborates the conclusion that although some beta cells are present in the pancreata of the diabetic streptozotocin-treated C57BL/6 mice, these are very rare (S6). Overtly diabetic AAV2/8-HLP-hINSco -treated NOD mice have no endogenous beta cells left in their pancreata while glucagon-positive alpha cells remain, and a few infiltrating T cells can still be seen in the surrounding tissue (Fig. 4a, bottom panels). Importantly, T cells can also be detected in the livers of diabetic AAV2/8-HLP-hINSco-treated NOD mice (Fig. 4b, right) (5.2 ± 1.24 per liver section *n* = 5), but not in the treated C57BL/6 mice (Fig. 4b, left). These T cells consist mainly of CD8^+^ T cells (Fig. 4c) (3.6 ± 0.51 per liver section *n* = 5). The reactivation of insulin-specific T cells was detected with IFN-γ ELISPOT revealing that there were significantly more IFN-γ-producing CD8^+^ T cells in diabetic AAV2/8-HLP-hINSco-treated NOD mice than in untreated NOD mice both at 30 days (Fig. 4d, bottom panels) and 200 days (Fig. 4e, bottom panels), while the CD8^+^ T cells from C57BL/6 mice produced negligible levels of IFN-γ in response to insulin peptide both before and after treatment at all time points (Fig. 4d, e, top panels). Cells from both mouse strains responded well to polyclonal activation with PMA and Ionomycin (S7). Interestingly, NOD mice not only demonstrated a more vigorous response to insulin, but also to peptide from the AAV2/8 vector itself (Fig. 4e, bottom right panel), indicating that NOD mice have more easily triggered immune responses in general. This hypothesis is further supported by the fact that splenocytes from treated NOD mice produced significantly less of the immunosuppressive cytokine interleukin-10 (Fig. 4f) but more of the anti-AAV8 antibodies (Fig. 4g) than those from treated C57BL/6, and displayed an overall higher metabolic activity (S8).

Diabetic NOD mouse recipients of AAV2/8-HLP-hINSco required higher doses of vector, and had higher levels of serum human C-peptide on achieving euglycaemia than C57BL/6 mice (Figs. 1 and 2). Even when mostly restored, previously diabetic NOD mice displayed more fluctuating blood glucose readings, indicating a less stable supply of insulin (Fig. 2a). We wondered if the anti-insulin antibodies present in the serum of NOD mice (S9) could bind the transgenically produced human insulin, and thus contribute to a situation where the insulin is present but inactive. This phenomenon has been described in insulin-treatment naïve people presenting with hypoglycaemic episodes (described as insulin autoimmune syndrome (IAS) or Hirata disease [26]) as well as in patients with labile diabetes treated with modern genetically engineered insulin analogues. Insulin bound by antibodies can lose its biological effect and its half-life increases from 4 to 6 min to several weeks. We used a mass spectrometry based peptidomics analysis of serum after acetonitrile precipitation [27], which allows release of any bound insulin from antibodies, to assess whether the treated NOD mice had more transgenic human insulin in their serum. The peptidomics approach identified peptides derived from the human insulin transgene circulating in the plasma of AAV2/8-HLP-hINSco-treated mice, in particular the C-peptide (S10A), which showed the highest signal. These transgene derived peptides were only detected in the AAV2/8-HLP-hINSco-treated mice. We could detect peptides derived from both the A- and B-chain of human insulin in both mouse strains, with no significantly higher abundance in NOD mice, suggesting no antibody-mediated serum insulin concentration increases were in effect (S10B).

### Suppression of adaptive immune responses improves the effect of AAV2/8-HLP-hINSco in naturally diabetic NOD mice

**Fig. 5:**
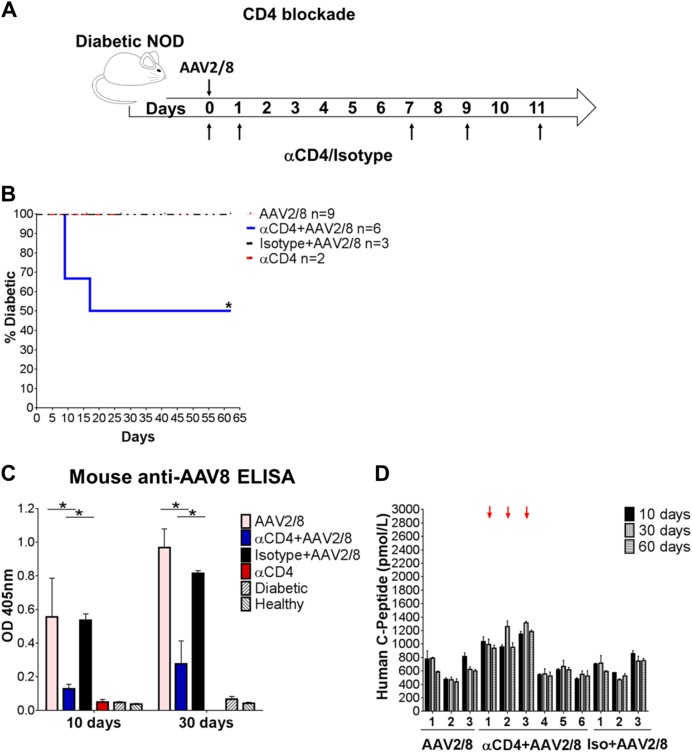
Dampening the immune response improves therapeutic efficacy of gene therapy in naturally diabetic mice. **a** Established diabetic NOD mice were treated with either the IgG2a isotype control (MAC219), or a non-depleting antibody to CD4 (YTS177), or a combination of anti-CD4 and 25 × 10^9^ vg/mouse AAV2/8-HLP-hINSco (indicated as AAV2/8). **b** Blood glucose measurements for each experimental mouse were taken from the day of AAV2/8-insulin vector injection and are represented as average in a survival curve showing the percentage of diabetic mice. *(*p* ≤ 0.05), log-rank survival test. **c** Blood plasma of experimental mice tested for anti-AAV8 antibodies by ELISA. The absorbances at 405 nm correlate with the concentration of the antibody. *(*p* ≤ 0.05), unpaired Student’s *t*-test. **d** Human C- peptide levels of individual experimental mice (numbered 1–6) at day 10, 30 and 60 from the day of AAV2/8-HLP-hINSco injection. Arrows indicate mice responding to the CD4 blockade/AAV2/8-insulin combination therapy. Data shown are expressed as mean ± SE

Injection of non-depleting anti-CD4 antibody YTS177 can suppress adaptive immune responses and induce immunological tolerance to islet antigens and AAV capsid [28], but if administered alone cannot reverse overt spontaneous diabetes in NOD mice [29–31]. To determine whether suppression of adaptive immune responses could increase the efficacy of the vector, we investigated whether co-treatment with YTS177 affected restoration of euglycaemia (Fig. 5a). Fifty percent of the diabetic NOD mice injected with low dose (25 × 10^9^ vg/mouse) AAV2/8-HLP-hINSco, and also treated with repeated injections of YTS177, achieved reversal of diabetes (here defined as blood glucose levels decreasing to below 13 mM), while none of the mice treated with isotype control antibody, vector alone or anti-CD4 antibody alone did (Fig. 5b). The CD4 blockade clearly decreased adaptive responses, as the production of anti-AAV8 antibodies was significantly decreased in YTS177-treated mice (Fig. 5c) and human C-peptide levels increased in those mice responding to the combination therapy (Fig. 5d, red arrows).

### Efficacy of AAV2/8-HLP-hINSco in NOD mice is inhibited by vector specific immunity

**Fig. 6:**
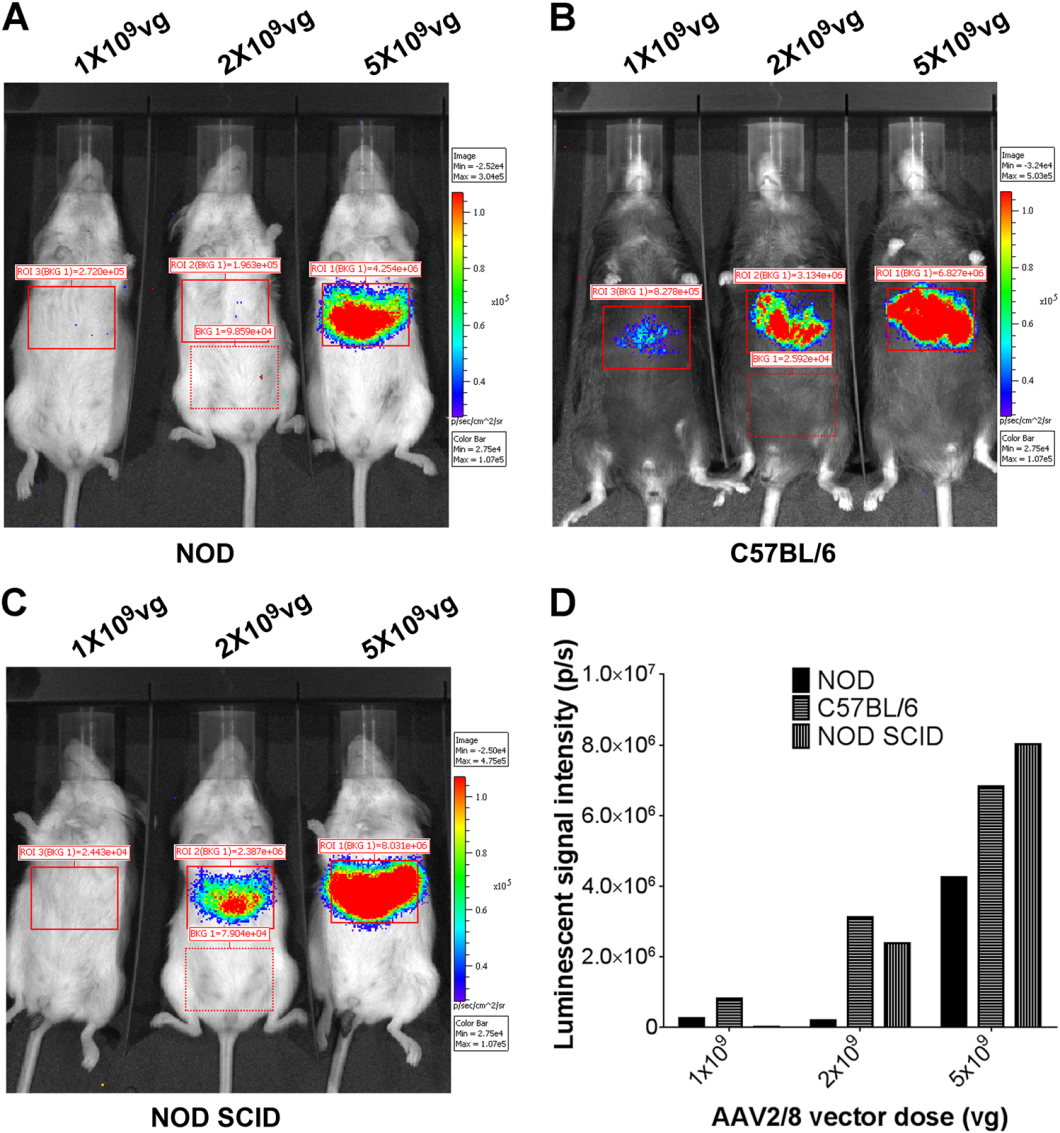
Low transduction levels in NOD mice infected with AAV2/8-insulin therapy are not specific to the insulin transgene. NOD (**a**), C57BL/6 (**b**) and NOD^*scid*^ (**c**) mice were injected i.v. with different doses of ssAAV2/8-HLP-Luciferase (indicated as AAV2/8). After 10 days, mice were injected i.p. with luciferin, anaesthetized and measured for live luminosity by an image capturing device (IVIS). Quantification of luminescent intensity (photons/second) for the boxed areas in **a**–**c** with background signal deducted (**d**)

The anti-CD4 treatment showed that adaptive immune responses could prevent the efficacy of the vector, but it did not specify whether the inhibiting immune responses were specific for insulin in particular. To elucidate this, we used a different vector, which expressed luciferase rather than insulin, allowing the transduction efficacy to be imaged in vivo at different time points after injection using whole-body imaging. If the inhibition seen in diabetic mice had been insulin-specific, the efficacy of this luciferase-containing vector should have been equally high in NOD mice (Fig. 6a), C57BL/6 mice (Fig. 6b), and NOD^*scid*^ mice (Fig. 6c). However, even with this vector we observed much lower expression in the immunocompetent healthy NOD mice than healthy C57BL/6 mice (Fig. 6a, summarised in Fig. 6d), indicating that although insulin-specific responses are reactivated in the vector-treated NOD mice, with accompanying T cell infiltration into the liver, the blocking immune responses also have other targets.

**Fig. 7:**
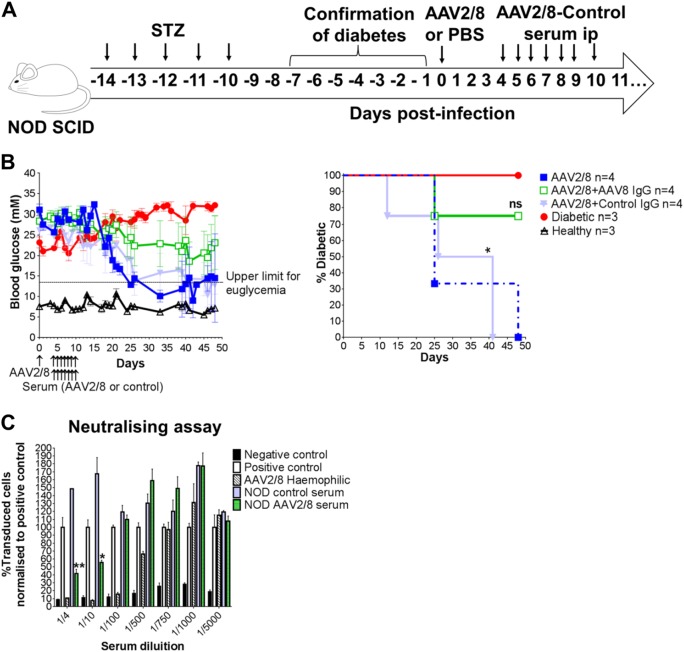
Anti-AAV2/8 passive immunisation diminishes the AAV2/8-insulin-mediated therapeutic effect in diabetic NOD^*scid*^ mice. **a** NOD^*scid*^ male mice rendered diabetic with streptozotocin (40 mg/kg i.p. for 5 days) were treated with 5 × 10^9^ vg AAV2/8-HLP-hINSco (indicated as AAV2/8) (day 0). The serum of immunocompetent NOD mice either injected with 5 × 10^9^ vg or vehicle only was injected i.p. into the NOD^*scid*^ mice 4 days after the beginning of the therapy and for the following 7 days. The start date of passive immunisation was temporally determined by the appearance of anti-AAV8 antibody response in the immunocompetent NOD mice via ELISA. Blood glucose levels of treated animals monitored over time represented both as average measurements ± SE (**b**, left) and as a resulting percentage of diabetic mice (**b**, right). Statistical difference measured against diabetic control. Ns =  non significant, *(*p* ≤ 0.05), log rank survival test. **c** NOD serum used to passively immunise AAV2/8-insulin-treated NOD^*scid*^ has AAV2/8 blocking capabilities conferred by anti-AAV2/8-neutralising antibodies. Percentage of cell transduction in an in vitro viral neutralising assay. Number of transduced cells was normalised to positive control. AAV2/8 haemophilic represents a serum sample coming from an AAV2/8-treated dog known to have developed immunity against the vector. Statistical difference compared to positive control, *(*p* ≤ 0.05), **(*p* ≤ 0.01), two-way Anova

To further test whether antibody responses to AAV vector or insulin could block effective transduction with the vector, we performed an antibody transfer experiment. We rendered NOD^*scid*^ male mice diabetic with streptozotocin, and then treated them with 5 × 10^9^ vg/mouse, the dose that had previously demonstrated complete restoration of euglycaemia in immunodeficient NSG mice [7] and NOD^*scid*^ mice (S1). NOD^*scid*^ mice have a complete lack of an adaptive immune system and very limited generation of antibodies [32]. We performed serum transfer every day from day 4 (the earliest time point in immunocompetent NOD mice when AAV8-specific antibodies can be detected after infection) to day 10 after AAV2/8-HLP-hINSco treatment, with either AAV2/8-HLP-hINSco-exposed serum or control NOD serum (Fig. 7a). We found that the 5 × 10^9^ vg/mouse dose restored euglycaemia in diabetic NOD^*scid*^ male mice just as we had observed previously. In addition, we found that serum from AAV2/8-HLP-hINSco-exposed NOD mice blocked restoration of euglycaemia in the diabetic NOD^*scid*^ males, while control NOD mouse serum did not (Fig. 7b, left and right panels). The AAV2/8 vector exposed serum was harvested from exposed NOD mice 9 days after injection, a time point when a strong antibody response has been established. The transferred antibodies were also able to block transduction of cells with vector coding for GFP in vitro (Fig. 7c).

## Discussion

We investigated whether an AAV2/8 vector expressing furin cleavable insulin under the liver-specific promoter (HLP), which can restore euglycaemia in streptozotocin-induced diabetic immunocompromised mice [7], is also effective in immunocompetent streptozotocin-induced diabetic mice, and in spontaneously diabetic non obese diabetic (NOD) mice. We also investigated the occurrence of immune responses to the vector and the transgene in each of these conditions. We find that AAV2/8-insulin gene therapy can indeed correct hyperglycaemia in both of these models of diabetes, but that the dose requirements for success in autoimmune, spontaneously diabetic NOD mice is 7–10-fold higher than in non-autoimmune C57BL/6 mice. This is not due either to increased insulin resistance in the NOD mice, or to differences between male and female mice. Diabetic NOD mice have established T cell responses to insulin, and these are reactivated by the introduction of insulin expression in the liver cells. The epitope studied, InsB15-23 (LYLVCGERG), is conserved between mouse- and human insulin [33]. We see evidence of immune infiltration in the livers of vector-treated NOD mice, accompanied by increased activation of insulin-specific CTL; however, the mice treated with the higher doses of vector retain high and stable levels of human C-peptide in the serum. We hypothesised that anti-insulin antibodies may contribute to a need for higher insulin secretion by neutralising the released insulin product, a condition known as Hirata’s syndrome. We utilised a novel mass spec-based technique to assess the presence of antibody–insulin complexes in the serum of AAV2/8-HLP-hINSco-treated NOD mice, but found no evidence of this. However, anti-inflammatory treatment with non-depleting CD4^+^ T cell antibody increased the efficacy of the AAV2/8-insulin gene therapy treatment, allowing restoration of euglycaemia using a lower dose that was not effective in isotype antibody-treated diabetic control NOD mice. Furthermore, transfer of AAV2/8-HLP-hINSco-reactive serum from previously treated NOD mice also decreased the efficacy of the treatment in NOD^*scid*^ mice, indicating that both T cell and B cell mediated responses act as barriers to AAV2/8-insulin gene therapy success.

Our data indicate that there are barriers to effective AAV2/8-insulin gene therapy in autoimmune NOD mouse recipients, and that these are caused both by reactivation of anti-insulin immune responses as well as a generalised higher activation state of the NOD mouse immune system. We saw reactivation of anti-insulin-specific CD8^+^ T cells, and infiltration of T cells in the liver where the insulin transgene is expressed, but although these responses almost certainly contribute to the prevention of effective AAV2/8-insulin therapy, it is also clear that vector-specific immune responses play a major role in NOD mouse resistance. In order to counteract the immune mediated destruction of insulin or insulin-producing cells, it is plausible that higher circulating levels of transgene are required to allow stable supply of insulin and therefore proper glycaemic control.

NOD mice have a deficiency in the inhibitory Fc receptor FcγRIIb, leading to greater production of antibody after stimulation [34, 35]. We show that the autoimmune NOD mice generate higher titres of anti-AAV antibodies, and our serum transfer experiments demonstrate that these anti-AAV antibodies can block efficacy of the treatment. However, immunosuppression with cyclosporine [28] or non-depleting anti-CD4 antibody [28] (and data presented in this report) can inhibit the priming or reactivation of immune responses.

A strong antibody response to the AAV2/8 vector was observed in immunocompetent mice only after 9 days from the day of gene therapy administration. At that point it is plausible to infer that the transduction of most targeted cells had already occurred as well as the expression of transgenic insulin. In the absence of cellular immune response towards the vector or the transgene, therapeutic efficiency could be long-lasting and negatively impacted only by the turnover rates of transduced cells. It is still not clear how long AAV particles can be detected in vivo and the effect that this could have on cellular immunity in the long term. However, our data seem to suggest that in NOD mice AAV2/8 capsids gain access to the MHC class I antigen presentation pathway and trigger capsid-specific CD8+ T cell responses. This, in combination with the enhanced transgene immunogenicity and possibly robust innate immune recognition of AAV, could induce the destruction of transduced hepatocytes. This phenomenon was observed only in the livers of the treated NODs, not in the C57BL/6 mice. The enhanced immunogenicity of vector and transgene in the NOD mice could be promoted by an environment less immunologically tolerant, a result also of the different microbiome composition [36, 37]. The therapeutic high dose of vector used for the NOD mice could also exacerbate the deleterious immune response already seen with lower doses.

An important observation is the very high efficacy of the AAV2/8-mediated insulin gene therapy treatment in NOD^*scid*^ mice, which are immunodeficient due to a deletion in the DNA repair gene *Prkdc* that prevents successful recombination of T- and B cell receptors [32]. The gene product from this gene, DNA-PK, which is also a component of the system that senses foreign nucleotides in the cell to initiate innate immune responses [38] has been reported to be important for successful infection with AAV viruses [39], raising the possibility that the big difference in dose requirement between diabetic NOD mice and diabetic NOD^*scid*^ mice may be due to the effects of this gene on innate immunity in addition to effects on adaptive immunity.

The liver itself has tolerogenic properties, due to a microenvironment characterised by low over all MHC class I expression and an abundance of particular antigen-presenting cells [40]. Transplant recipients who receive a combined kidney and liver transplant have lower rejection rates and sustain better glomerular filtration 5 years after transplant than recipients of kidney transplant alone [41]. This may in part be caused by tolerogenic properties of antigen presentation within the liver, particularly by the liver-resident Kupffer cells [42] which can expand the population of regulatory T cells [43]. Previous studies have shown that AAV-mediated expression in the liver can facilitate immune tolerance to clotting factors in mice [44], and, perhaps of greater importance to our present study, lentiviral vector-mediated liver-specific expression of the immunogenic insulin peptide InsB15-23 can induce insulin-specific Treg-mediated immune tolerance in NOD mice [45]. It is possible that the sporadic nature of the immune infiltration in the livers and the longevity of transgenic insulin expression in AAV2/8-insulin-treated NOD mice, in spite of the recorded reactivation of effector immune cells, reflects a similar induction of immune tolerance.

Secretion of sequestered insulin from beta cells is regulated by levels of surrounding glucose and signals induced by incretins from the gut. If too much insulin were secreted with insufficient glucose around there is a risk of hypoglycaemia, as we found when titrating the vector dose, which can be lethal. This is a serious concern when considering clinical application of insulin gene therapy. Several approaches have been suggested to solve this, for example, use of glucokinase to increase uptake [13], controlled sequestration in the ER [46] and the use of S14-based glucose-inducible regulatory elements [16], but it remains difficult to imagine a gene therapy approach that could fully imitate the efficient and sensitive responses and release mechanisms of the natural beta cell. In fact, it may be detrimental to devise a glucose responsive system which reacts to glucose levels several hours in the past, as a promoter-based system would. An alternative approach is to support the existing beta cell population rather than replace it by targeting insulin-like growth factor 1 (IGF1) expression to the beta cells using gene therapy. This approach has yielded positive results in NOD mice [47]. Regardless of which system is ultimately chosen, identifying the immunological barriers to treatment and strategies to overcome them will be of central importance for achieving success.

For most monogenic disorders, the approach commonly adopted to reduce or avoid AAV-capsid-specific humoral and cellular responses upon gene therapy treatment, is the administration of immune-suppressive drugs. As shown by McIntosh et al. [28] and Han et al. [48], transient immunosuppression achieved at the time of vector administration allows successful attenuation of antibody response to the vector and the transgene, with increased transduction efficiency, in models of haemophilia B and Pompe diseases. Importantly, this strategy allows also for vector readministration, which is particularly relevant for chronic disorders where the therapeutic effect of one round of infection may not be life long due to changed protein level requirements and/or turnover rates of the transduced cells. In our study we showed that the combination of an immunomodulatory agent such as the non-depleting anti-CD4 antibody with AAV-based insulin gene therapy allowed increased therapeutic benefit also for Type 1 diabetes. This could be the way forward also for human autoimmune diabetes, should the AAV-based treatment be taken to the clinic. As general immunosuppression protocols are associated with considerable side effects, such as increased susceptibility to infection [49] as well as reactivation of dormant infections [50], it is important to weigh up the risk–benefit ratio for any given approach. Further pre-clinical investigations could help to identify safe and specific transient capsid-derived antigen immunomodulators that would circumvent the risks associated with systemic immunosuppression. These pharmaceutical agents would ideally still allow for induction of robust long-term peripheral tolerance to the transgenic insulin and would not affect transduction efficiency.

In conclusion, AAV2/8-mediated liver-specific insulin gene therapy is inhibited by both insulin- and vector-specific immune responses in autoimmune diabetic NOD mice, but efficacy and long-lasting expression of transgenic insulin can be achieved by increasing the dose of vector and by administering immunosuppressive treatment. Despite raising a modest anti-AAV2/8 antibody response, immunocompetent C57BL/6 mice respond well to the treatment. This, in agreement with a previous study of immunocompetent CD-1 mice rendered diabetic with streptozotocin [15], indicates that the presence of a functional immune system does not represent an impediment to the success of the AAV-mediated gene therapy per se, but that particular qualities of the recipient immune response dictate the outcome. This could be important when considering clinical application. Furthermore, low-dose-treated diabetic NOD mice, which failed to return to normoglycaemia, continue to gain weight and appear well, suggesting that even low doses of insulin gene therapy vector can have beneficial effects while avoiding the risk of causing hypoglycaemia. Our data suggest that low expression of transgenic insulin could function as a complement to insulin injections, and has the potential to improve quality of life in people with diabetes.

## Methods

### Mice

NOD and NOD^*scid*^ mice were bred at the Department of Pathology, University of Cambridge. C57BL/6 male mice were purchased from Envigo and entered experimental protocols between 6 and 10 weeks of age. The mice were housed in individually ventilated cages and kept under controlled light (12 h light/dark cycles), temperature, and humidity conditions. They had free access to water and food, unless otherwise stated. This study was carried out in accordance with UK Home Office regulations and approved by the University of Cambridge Animal Welfare and Ethical Review Body.

### Chemical induction and confirmation of diabetes

NOD, NOD^*scid*^ and C57BL/6 male mice were injected intraperitoneally for five consecutive days with Streptozotocin (STZ, Sigma-Aldrich, 40 μg/g body weight) dissolved in citrate buffer (pH 4.2). No loss of mice occurred as a result of the STZ treatment. Diabetes normally developed within 10–14 days [51]. Eighty per cent of NOD female mice naturally developed diabetes between 12 and 30 weeks of age.

Body weight was measured and blood glucose tested via the tail vein with a glucometer. Mice were considered diabetic when blood glucose levels were >13.5 mM on three successive days as determined by a Contour Next blood glucose monitoring System (Bayer). Established diabetic mice were randomly divided into different groups of similar sizes, mixed between different cages. Power calculation to determine group sizes were performed using the www.dssresearch.com online resource. The investigators were not blinded to the group allocation or analysis; however, the readout consisted of objective blood glucose measurements not affected by the operators’ expectations. Any treated or control mice approaching loss of 15% of original body weight or otherwise showing signs of reduced wellbeing were culled in accordance with Home Office regulations. Long-lasting monitoring of treated mice was performed for as long as allowed by the Home Office project licence under which this work was carried out.

### Gene therapy treatment

ssAAV2/8-HLP-hINSco vector (co stands for codon optimised) encoding the furin cleavable human proinsulin gene [52] was designed, assembled, purified and titrated as previously described [7]. The production of the vector was either performed at UCL or commissioned from the Penn Vector Core Facility (University of Pennsylvania). Confirmed diabetic mice were injected intravenously with either the indicated doses of AAV2/8-HLP-hINSco or an equivalent volume of saline solution as per controls. Body weights and blood glucose levels were monitored on a daily basis from the beginning of the therapy. Blood, sampled from the lateral tail vein at the indicated time points, was collected in lithium-heparin tubes (VWR International) for plasma separation and then analysed for human C-peptide production on a Diasorin Liaison XL automated immunoassay analyser using a one-step chemiluminescence immunoassay (Kit No. 316171) in the Cambridge Biochemical Assay Laboratory, Addenbrooke’s Hospital. All reagents, standards and consumables are those supplied by the manufacturer.

### Assessment of impaired glucose metabolism

Treated and control animals were subjected to fasting by withdrawing the food from the cages for 16 h while still providing access to water.

To determine the sensitivity of insulin receptors, experimental mice were subjected to insulin tolerance tests. Mice were fasted for 6 h with access to water and were then injected intraperitoneally with 0.375 μU human insulin (Lilly)/g body weight and tested for blood glucose at 0, 15, 30, 60 and 120 min after injection. Results were then normalised to baseline fasting glucose.

To assess insulin production in response to glucose challenge, the mice were fasted overnight, then glucose (1 mg/g of body weight, in PBS) or vehicle alone was injected s.c. in the scruff of the neck. Blood was drawn after 15 min, and serum prepared and analysed for mouse insulin using the Mesoscale assay (MSD) at the Cambridge Biochemical Assay Laboratory, University of Cambridge.

### In vitro AAV neutralisation assay

293T cells were obtained from the American Type Culture Collection (ATCC, VA, USA) maintained in Dulbecco's modified Eagle's medium (DMEM) (Sigma) supplemented with 10% foetal calf serum (FCS), 1% glutamine and 1% penicillin/streptomycin at 37 °C and 5% CO_2_. 2 × 10^4^ cells/well in complete medium were seeded into 96-well plates the morning of infection. The mouse sera to test were heat-inactivated at 56 °C for 1 h and diluted in DMEM with concentrations ranging from 1/4 to 1/5000. Wells with the same volume of diluted FCS served as controls. 2 × 10^8^/well viral genomes of scAAV2/8-CMV-GFP vector diluted in an equal volume of DMEM were added to the diluted mouse sera and to some of the diluted FCS sera (positive controls), incubated at 37 °C for 30 min and then added to the 293T cells 5 h after cell plating. Negative controls did not receive any virus. In order to evaluate levels of inhibition, a serum sample obtained from a haemophilic dog 65 days after AAV8-based therapy injection was used for the assay. Thirty-six hours after infection, the transduction efficiency was analysed by quantifying the number of GFP^+^ 293T cells via flow cytometry (FACSCalibur, BD, New Jersey, USA). The percentage of inhibition was calculated relative to positive FCS control samples.

### Immunoassays

Plasma samples from mice were tested for the presence of antibody against human insulin and AAV8 using an ELISA. Ninety-six-well plates were coated overnight with either scAAV2/8-CMV-GFP (5 × 10^8^ vg/well) or Human insulin solution (Sigma, 10 μg/ml) diluted in 0.1 M NaHCO_3_ (pH 9.2)_,_ or NaHCO_3_ only. Wells were washed with 0.05% PBST (PBS with Tween 20) and blocked with 6% bovine serum albumin (BSA)/PBST for 1 h at 37 °C. Plasma samples diluted 1/1000 in 2% BSA/PBST (or dilution buffer only as for assay control) were applied to the wells in duplicate for 2 h at 37 °C. Wells were washed with 0.05% PBST and biotin-conjugated goat anti-Mouse antibody was applied (Novex Life Technology, cat. no. A16070, 1:10,000 in 2% BSA/PBST) for 45 min at RT followed by Peroxidase-Avidin (Thermo Fisher Scientific, 1:3500) for 30 min at RT. ABST substrate (Thermo Scientific) was added to develop the colorimetric reaction. Anti-insulin and anti-AAV8 antibody titres correlate with absorbances at 405 nm. Assay and sample control values were deducted from final measurements.

The frequency of AAV8 capsid- or insulin-specific CD8^+^ T cells in mouse splenocytes was determined using an IFN-γ enzyme-linked immunoSpot (ELISPOT) assay (R&D systems) according to the manufacturer’s instructions. In brief, plates were blocked with RPMI media prior to loading 2 × 10^5^ freshly isolated splenocytes/well and then stimulated with 50 ng/ml PMA + 2 μg/ml Ionomycin or 5 μg/ml peptide in RPMI-1640 (Sigma) + 2mM l-glutamine (Invitrogen) + 10% (v/v) FCS (PAA) + 1% penicillin/streptomycin + β-mercaptoethanol. Peptides (synthesised by Cambridge Research Biochemicals) were AAV8 capsid-specific NSLANPGIA [53], C57BL/6 CD8^+^ T cell insulin-specific SLYQLENYCN, NOD CD8^+^ T cell insulin-specific LYLVCGERG. Stimulation was performed for 40 h at 37 °C. Spots were counted with an Elispot Reader (AID, Strassberg, Germany) with intensity >10 and size >20.

Soluble IL-10 cytokine production was tested in the supernatants of splenocytes cultured in RPMI-1640 (Sigma) + 2mM l-glutamine (Invitrogen) + 10% (v/v) FCS (PAA) + 1% penicillin/streptomycin + β-mercaptoethanol with 50 ng/ml PMA + 2 μg/ml Ionomycin for 48 h at 37 °C. The amount of IL-10 protein was measured using a DuoSet® mouse IL-10 ELISA (R&D) according to the manufacturer’s instructions.

### In vivo bioluminescent imaging

Mice injected intravenously with ssAAV8-HLP-Luciferase at the indicated doses were monitored non-invasively for the whole-body distribution of the transgene on day 10. Mice were injected intraperitoneally with 2 mg/mouse XenoLight D-Luciferin (Perkin Elmer, Waltham, USA), anesthetised with isofluorane and imaged 15 min later using the IVIS Spectrum pre-clinical imaging system (Perkin Elmer, Waltham, USA). The bioluminescence was quantified from ROIs with deducted background signal using the Living Image 3.2 In Vivo Imaging Software (Perkin Elmer).

### Determination of viral genome copy numbers and insulin transcript levels in the mouse livers and pancreata

Livers and pancreata extracted from sacrificed mice were collected in RNAlater (Sigma) for organ preservation and stored at −20 °C. Total DNA and RNA were isolated using the All Prep DNA/RNA Mini Kit (Qiagen), according to the manufacturer’s instructions. Transgene copy number in the liver was determined using real-time PCR assay using Roto-Gene 3000 (Corbett Research, Sydney, Australia) and Rotor-Gene SYBR Green PCR Kit (Qiagen). The primers were designed against the HLP promoter (forward primer 5′-CAGGACGCTGTGGTTTCTG-3′ and reverse primer 5′-TGCCTGAAGAGAC-3′) and normalised with mouse GAPDH housekeeping gene (forward primer 5-′TGGAGAGCCCGCTCAGACCC-3′ and reverse primer 5′-GGATGGGGTGTCCCTGCGCC-3′) to obtain AAV genome copy/mouse cell. One microgram of total RNA was converted to cDNA using random hexamer primers (Integrated DNA Technologies), oligo-dT primers (Integrated DNA Technologies) and RevertAid™ H Minus Reverse Transcriptase (Thermo Fisher Scientific) according to the manufacturer’s protocol. Relative mRNA expression was quantitated by real-time qPCR. The primers were designed against the codon optimised human insulin (forward primer 5′-ACTGCTTCCTCTGCTTGCTC-3′ and reverse primer 5′-GCCTCTTTCTCCGCACACAA-3′) or mouse insulin 2 (mINS2) gene (forward primer 5′-CCACAAGTGGCACAACTGGA-3′ and reverse primer 5′-ACTGATCTACAATGCCACGCT-3′) and normalised against mouse 18S rRNA (forward primer 5′-CCTGCGGCTTAATTTGACTC-3′ and reverse primer 5′-CGCTGAGCCAGTCAGTGTAG-3′). All PCR reactions were performed in duplicate. The CT of each sample was obtained using RotorGene version 6.1 software. 2^−(ΔΔCT)^ was calculated and the relative expression levels were determined by arbitrarily assigning a value to one to controls.

### Passive immunisation

NOD female mice aged 8–30 weeks were injected intravenously with either PBS (control) or 5 × 10^9^ vg/mouse ssAAV2/8-HLP-hINSco and were blood sampled daily for testing occurrence of anti-AAV8 antibody via ELISA. Sera were collected 9 days post-injection by terminal cardiac puncture, pooled and tested for neutralising anti-AAV8 antibodies (see above). To passively immunise the immunocompromised NOD ^*scid*^, 0.4 ml of this pooled serum was injected intraperitoneally for 7 consecutive days 4 days after the beginning of the AAV2/8-HLP-hINSco gene therapy.

### CD4^+^ T cell blockade

Established diabetic NOD female mice were injected intravenously with 25 × 10^9^ vg/mouse of ssAAV2/8-HLP-hINSco (d0). Mice received 2 mg of either the IgG2a isotype control (MAC219), a non-depleting anti-mouse CD4 mAb (YTS177) as previously described [31] or PBS only injected 8 h prior to administering gene therapy (intravenously) and on days 1, 7, 9 and 11 (intraperitoneally). The YTS177 hybridoma was a kind gift from Professor Herman Waldmann, University of Oxford, and the MAC219 hybridoma was a kind gift from Professor Geoff Butcher, Babraham Institute. Both antibodies were produced in-house.

### Immunohistochemistry

Livers and pancreata removed from sacrificed mice were fixed in 4% PFA for 5 h at 4 °C, dehydrated in 30% sucrose overnight at 4 °C, and embedded in O.C.T mounting medium (VWR Chemicals) before freezing at −70 °C. Five micrometre cryostat (Leica) sections were air-dried, fixed in acetone for 6 min, air-dried, and stored at −20 °C. Immunolabelling was performed on sections pre-blocked with 1% BSA by incubating with rabbit anti glucagon (Millipore, cat no. AB932, 1:20) guinea pig anti-insulin (DAKO, cat no. A0564, 1:150), rabbit anti C-peptide (Cell Signalling, cat no. 4593S, 1:50), Rat anti-CD3 (Biolegend, cat no. 100202, 1:100), Rat anti-CD8 (Biolegend, cat no. 100702, 1:50) for 1 h at 4 °C. Incubation with relevant Alexa Fluor® secondary antibodies (Invitrogen, 1:500) and DAPI (4′,6-diamidino-2-phenylindole) (Invitrogen) followed. Sections were then mounted in Prolong Diamond Antifade solution (Thermo Fisher Scientific) and visualised using a Zeiss LSM 700 confocal microscope (Zeiss, Germany). Images were captured and processed using Zen software (Zeiss).

### Mass spectrometry analysis

Seventeen microliters of plasma were extracted with 100 µl of 80% acetonitrile in water and analysed as previously described [27].

### Mitochondrial activity

T cell oxygen consumption rate (OCR) was measured using the XF96 Analyzer (Agilent technologies, Santa Clara, California, USA). In brief, XF96-well plates were coated using Cell-Tak (BD Biosciences, San Jose, CA, USA) to allow T cell adhesion. T cells were isolated with the Mouse CD3^+^ T cell column enrichment kit (R&D) and plated at the concentration of 3 × 10^5^ cells/ 50 μl/well, and analysed using the Mitostress kit (Agilent technologies) according to the manufacturers’ instructions. Seahorse assay medium (Agilent technologies) was supplemented with the indicated glucose concentration, 1 mM glutamine and 1 mM pyruvate. Oligomycin was administered at 1.5 μM, FCCP at 1 μM and rotenone/Antimycin A at 1 μM (all from Agilent technologies).

### Statistical analysis

Statistical differences between experimental groups which had normal distribution and equal variances were determined by unpaired Student’s *t*-test using GraphPad Prism version 6.0 software (GraphPad, San Diego, CA). Data are presented as mean ± standard error (SE). Differences in incidence of diabetes between groups were assessed using the log-rank survival test. Significant *p*-values are indicated with ^Δ^, *(*p* ≤ 0.05), **(*p* ≤ 0.01), ***(*p* ≤ 0.001) or ****(*p* ≤ 0.0001).

## Supplementary information

Supplementary Figures
