# Supplementary material for: Immunosuppression overcomes insulin- and vector-specific immune responses that limit efficacy of AAV2/8-mediated insulin gene therapy in NOD mice
Source: Gene Ther. 2018 Dec 4;26(1):40–56. doi: 10.1038/s41434-018-0052-5 (PMC6514884; doi:10.1038/s41434-018-0052-5)
Supplement: Supplementary file 1 — Supplementary Figures [file 41434_2018_52_MOESM1_ESM.docx]

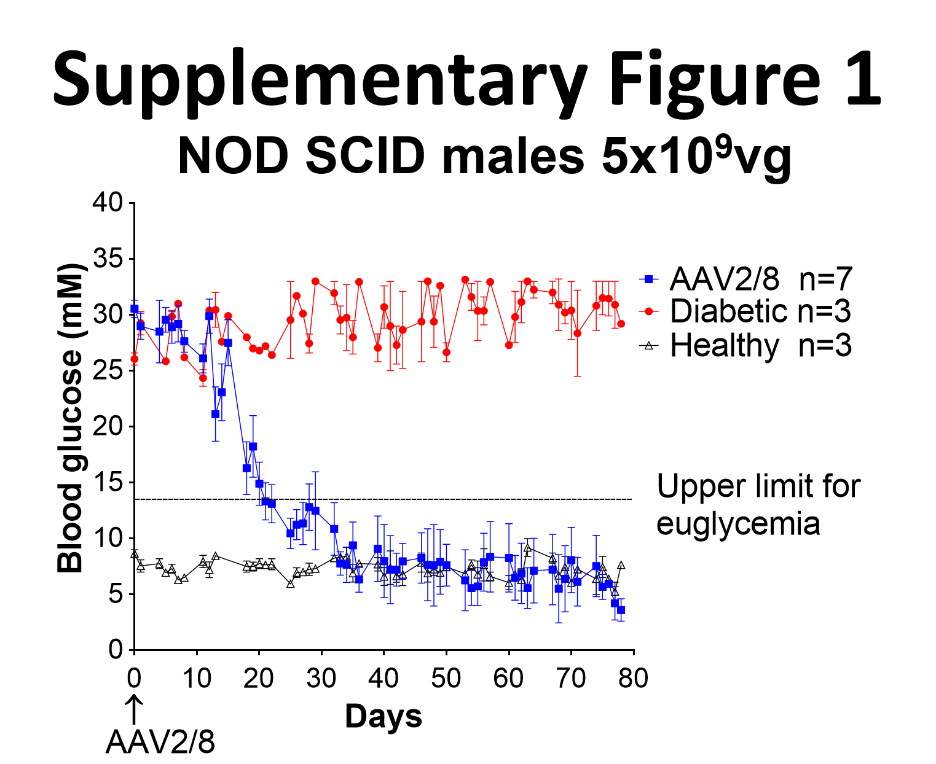


**Supplementary Fig.1: AAV2/8-HLP-hINSco administered at the dose of 5x10^9^vg /mouse confers control of diabetes in immunocompromised NOD*^scid^* mice.** NOD*^scid^* male mice, rendered diabetic with streptozotocin (40 mg/kg i.p. for 5 days), were treated with AAV2/8-Insulin therapy (indicated as AAV2/8) (day 0). Blood glucose measurements were taken after vector administration. Data shown are expressed as mean ± SE and are representative of 2 independent experiments.


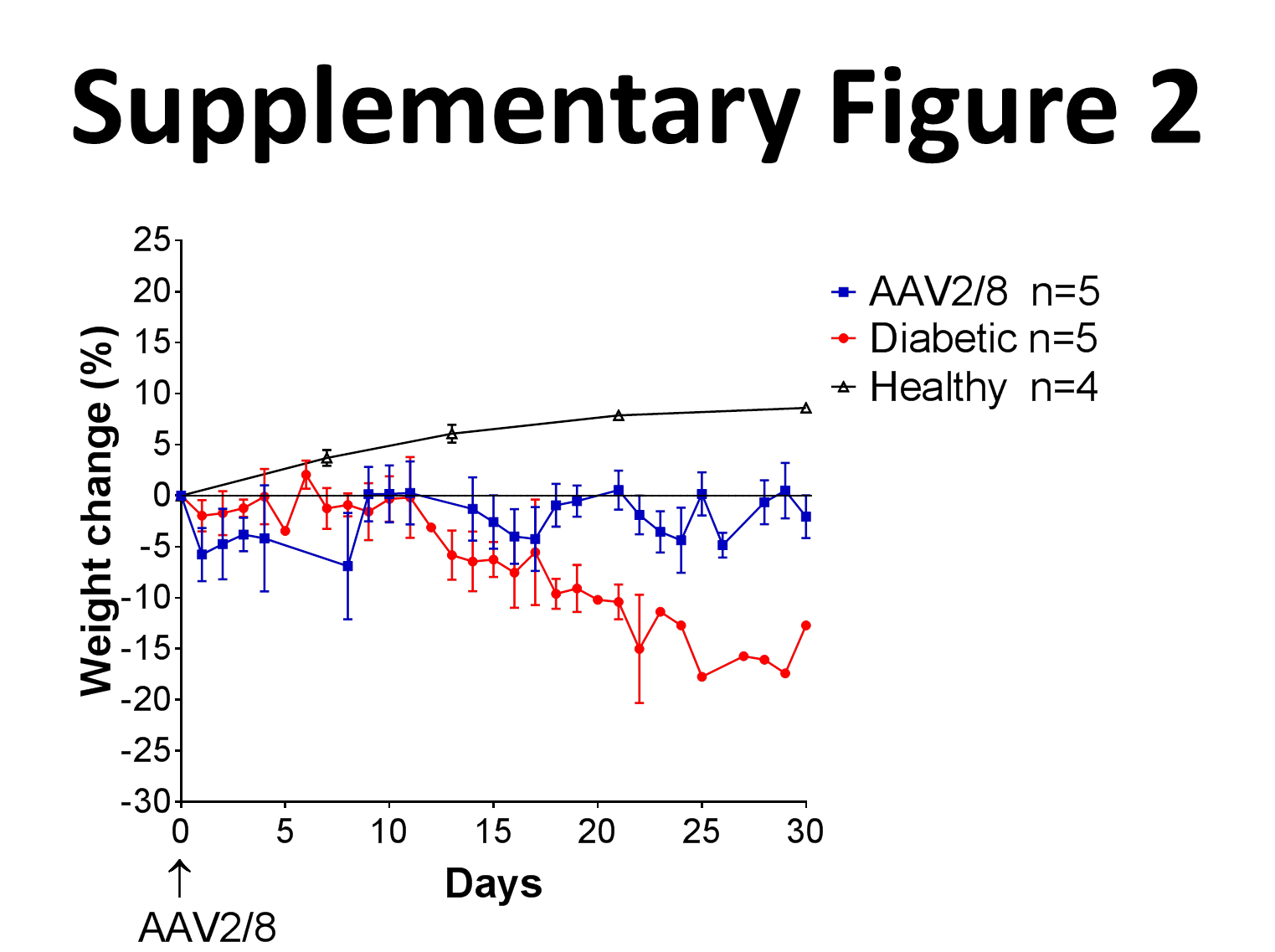


**Supplementary Fig.2: AAV2/8-HLP-hINSco administered at the dose of 5x10^9^vg /mouse allows maintenance of normal body weight in diabetic NOD mice.** Percentage of weight change from baseline over time for AAV2/8-Insulin –treated (indicated as AAV2/8), diabetic and healthy control NOD female mice. Data shown are expressed as mean ± SE and are representative of 2 independent experiments.


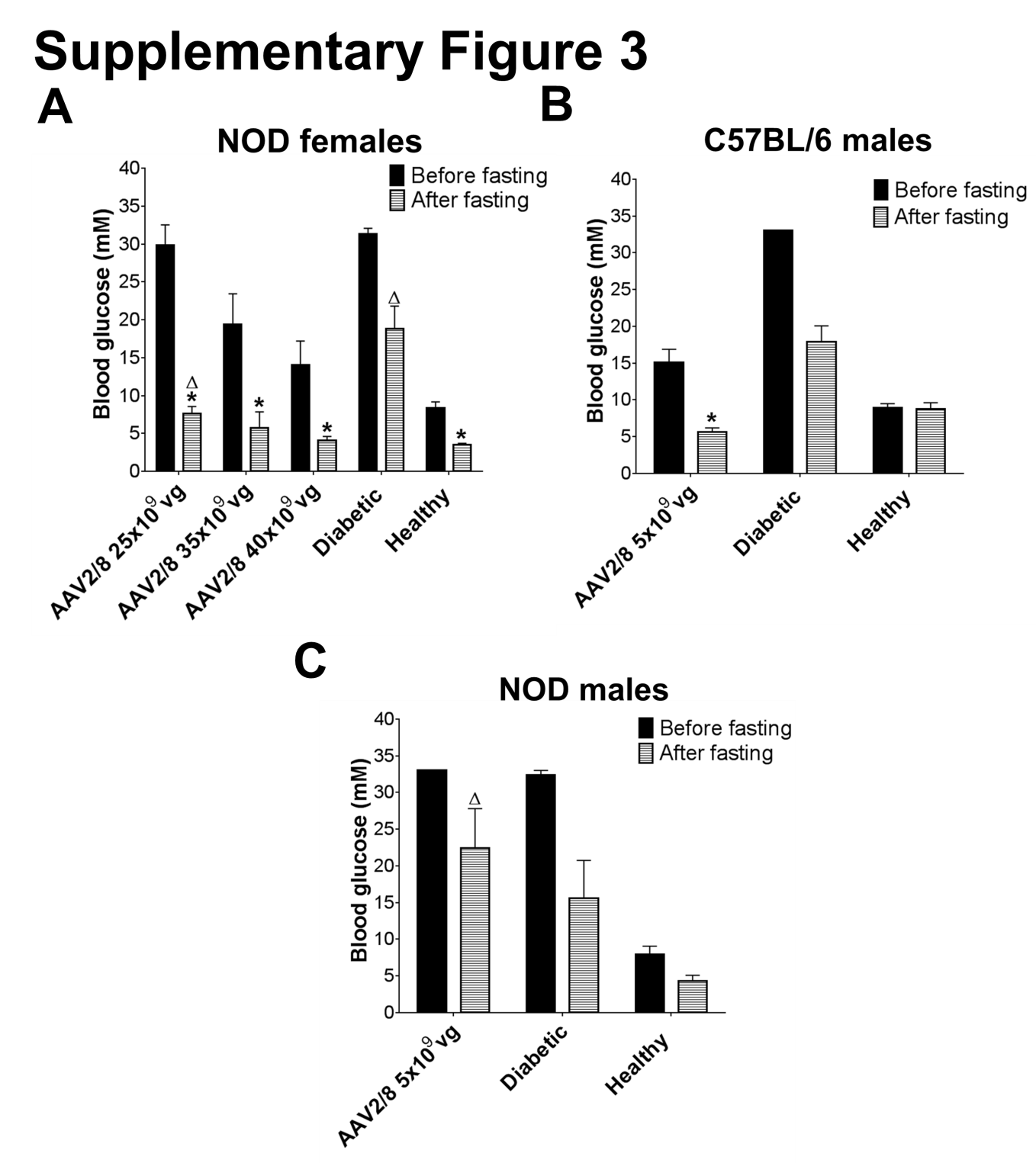


**Supplementary Fig. 3: Effect of fasting on blood glucose in AAV2/8-HLP-hINSco-treated diabetic NOD mice.** Blood glucose levels of AAV2/8-HLP-hINSco –treated (indicated as AAV2/8) and control NOD females (A), STZ-induced C57BL/6 male (B) and STZ-induced NOD male (C) mice were measured before and after 16 hour fasting. Results represented as average measurements ± SE based on at least 2 independent experiments (n=10). Statistical difference measured against corresponding healthy (Δ) or diabetic (*) controls. ^Δ,^* (p≤0.05), unpaired Student’s *t* test.


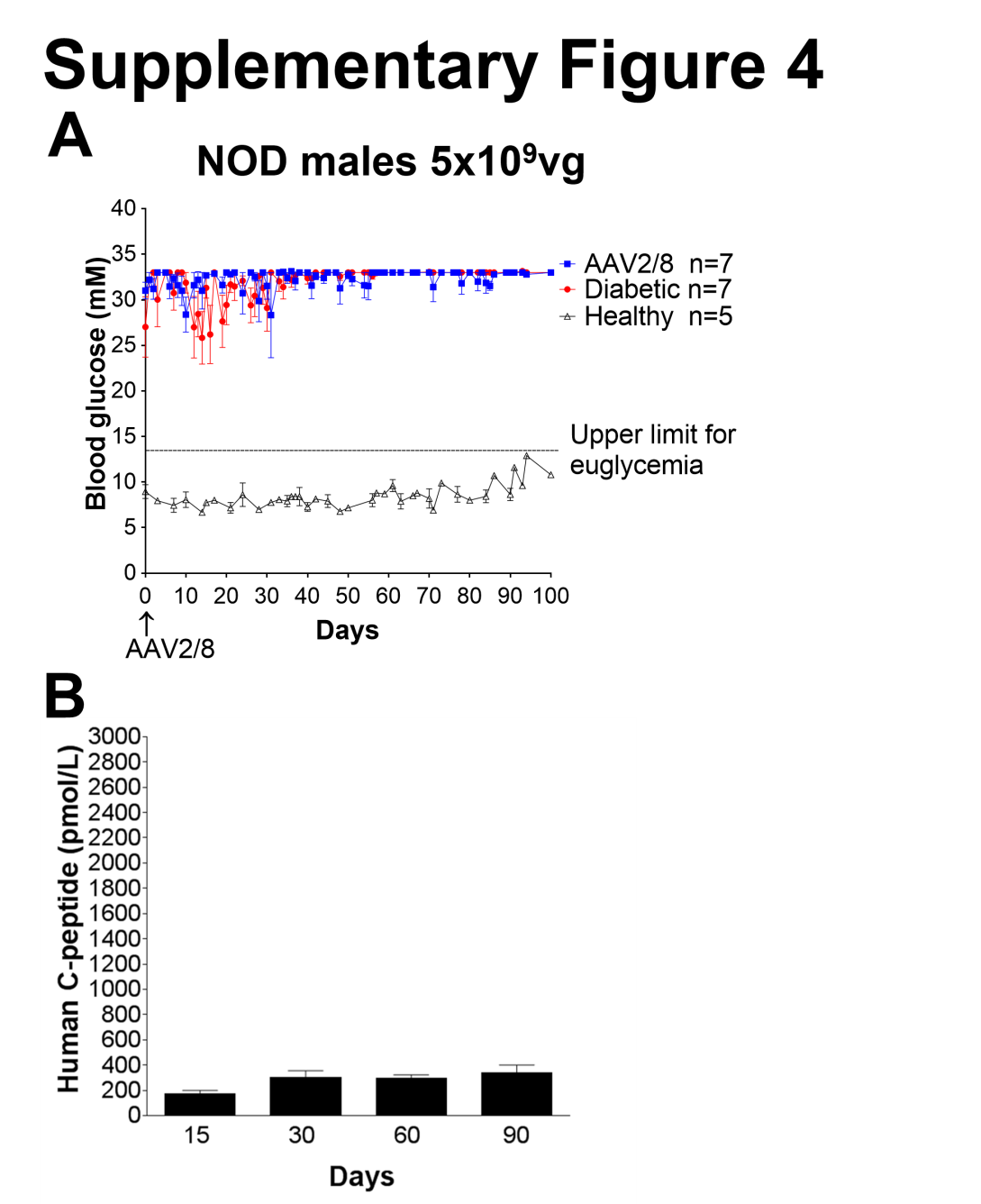


**Supplementary Fig. 4: AAV2/8-HLP-hINSco administered at the dose of 5x10^9^vg /mouse fails to show control of diabetes in chemically-induced diabetic NOD males.** NOD male mice, rendered diabetic with streptozotocin (40 mg/kg i.p. for 5 days), were treated with AAV2/8-HLP-hINSco (indicated as AAV2/8) (day 0). Blood glucose measurements (A) and human C-peptide levels (B) were measured after vector administration. Data shown are expressed as mean ± SE and are representative of 2 independent experiments.


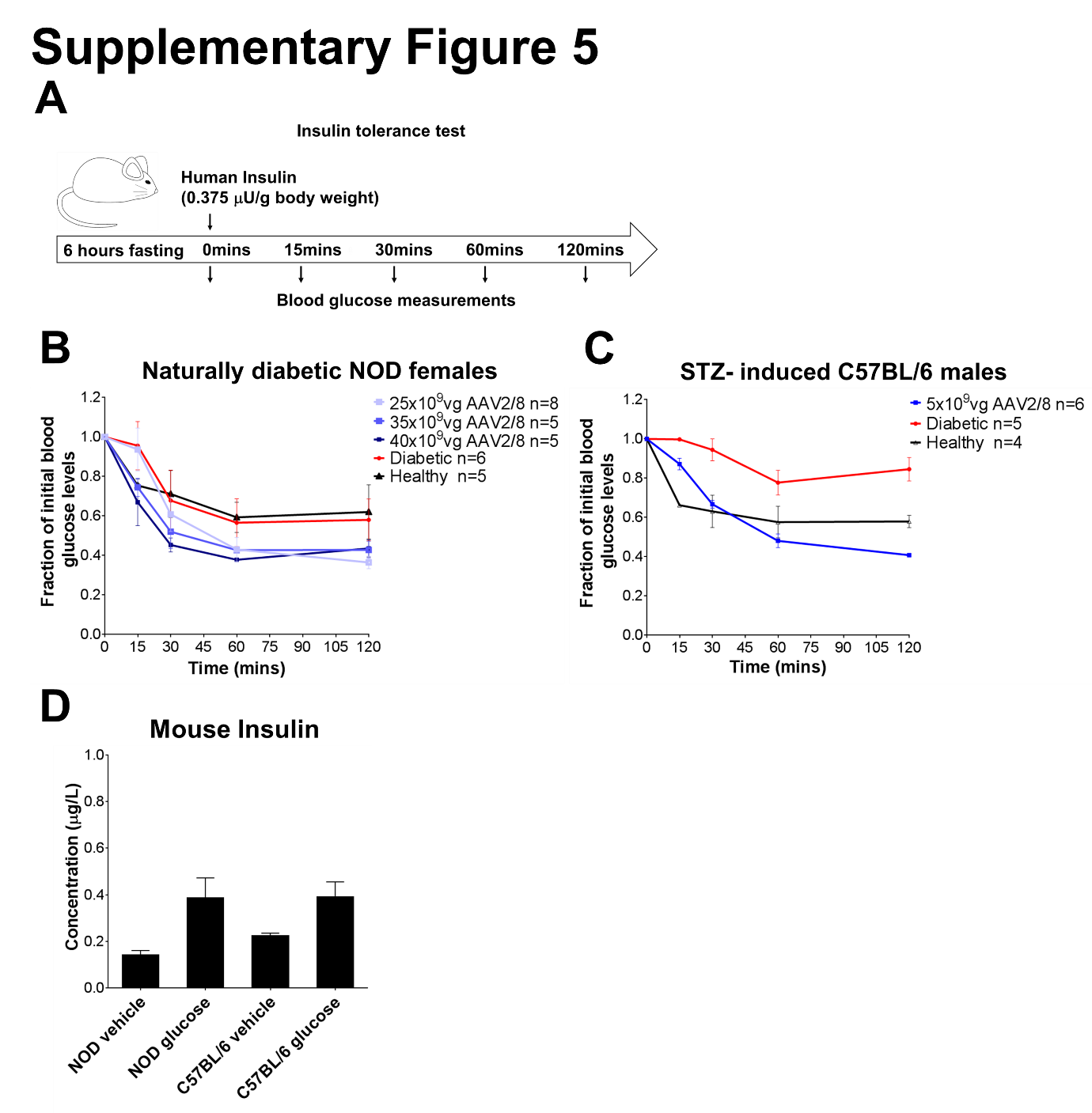


**Supplementary Fig. 5: Diabetic and AAV2/8-Insulin-treated NOD mice do not show impaired insulin tolerance.** Healthy, diabetic and AAV2/8-HLP-hINSco –treated (indicated as AAV2/8) mice were fasted for 6 hours, injected i.p. with 0.375μU human insulin/gram of body weight and monitored for blood glucose (A). NOD female (B) and C57BL/6 male (C) blood glucose measurements were normalized to baseline fasting glucose. (D) Levels of endogenous insulin levels in NOD and C57BL/6 mice before and after oral glucose challenge. Results represented as average measurements ± SE based on at least 2 independent experiments.


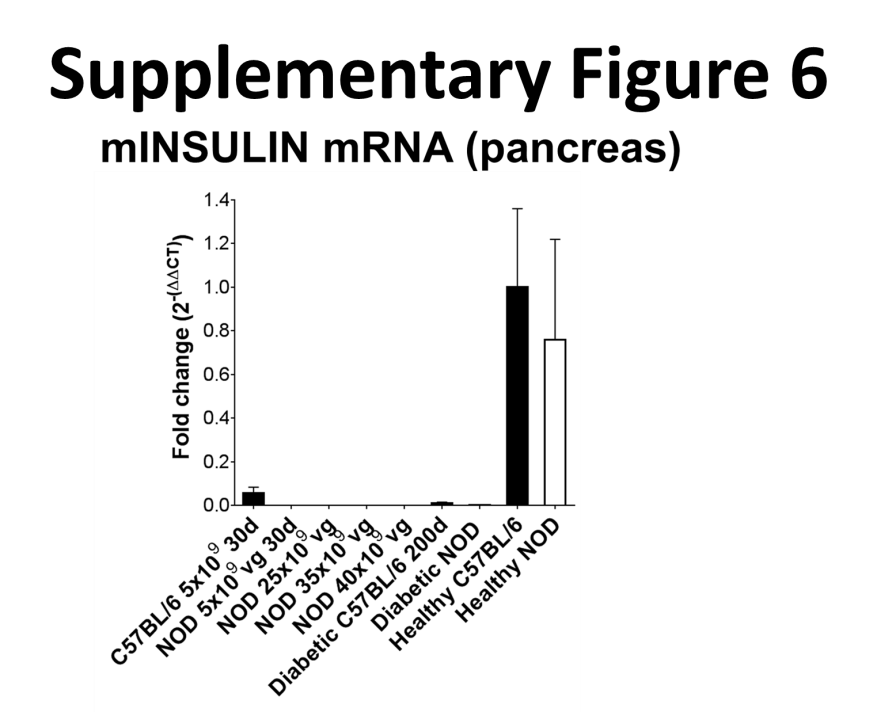


**Supplementary Fig. 6: Endogenous insulin levels produced by streptozotocin-treated C57BL/6 mice are negligible compared to healthy controls.** Endogenous mouse Insulin mRNA expression in the pancreata of NOD and C57BL/6 mice. Samples were analysed from animals that were either healthy, untreated diabetic or infected with AAV2/8-HLP-hINSco at the dose of 5x10^9^vg (30 days post- injection), or 25x10^9^vg, 35x10^9^vg, 40x10^9^vg (more than 200 days post- injection). Expression is relative to healthy C57BL/6 levels. Data shown are expressed as mean ± SE.


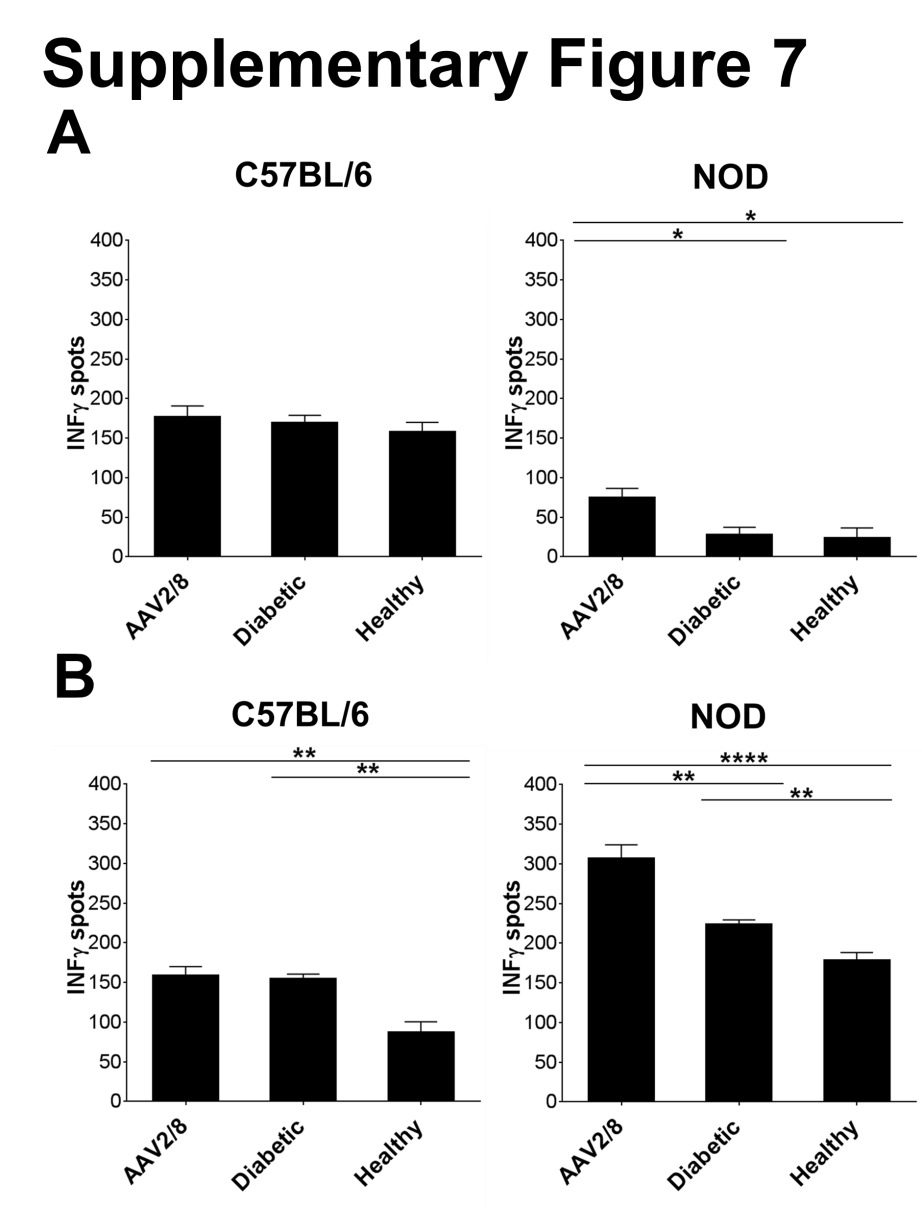


**Supplementary Fig. 7: *Ex vivo* stimulated splenocytes from AAV2/8-HLP-hINSco -treated NOD mice respond to polyclonal stimulation similarly to C57BL/6 controls as shown by IFN-γ ELISPOT.** Cells harvested from NOD and C57BL/6 mice after 30 days (A) or more than 200 days (B) from the day of the injection of the AAV2/8-Insulin vector (indicated as AAV2/8) were stimulated with PMA/Ionomycin as indicated in the methods and tested for INFγ production. * (p≤0.05), ** (p≤0.01), **** (p≤0.0001), unpaired Student’s *t* test. Data shown are expressed as mean ± SE and are representative of 2 independent experiments.


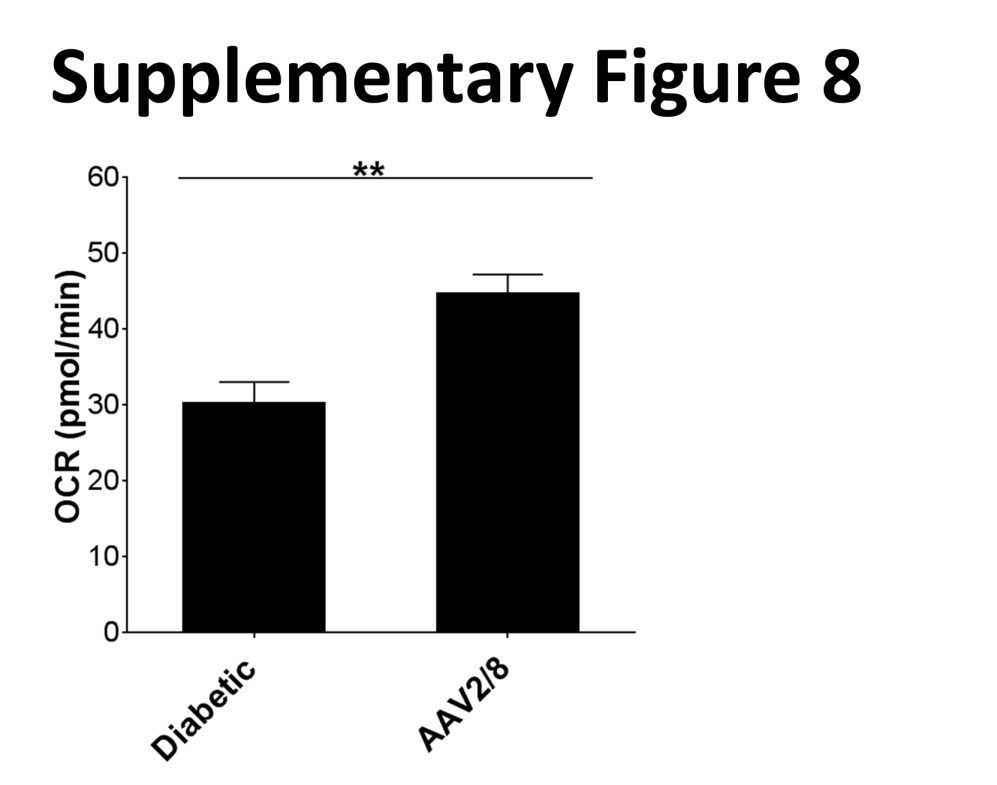


**Supplementary Fig. 8: T-cells of AAV2/8-HLP-hINSco -treated NOD mice are metabolically more active than diabetic controls.** Maximum mitochondrial respiratory capacity of *ex vivo* NOD female splenic lymphocytes after more than 200 days of treatment with therapeutic AAV2/8-HLP-hINSco dose (indicated as AAV2/8). Oxygen consumption Rate (OCR) was measured after the addition of the oxidative phosphorylation uncoupler drug FCCP. Data shown are expressed as mean ± SE. ** (p≤0.01), unpaired Student’s *t* test.


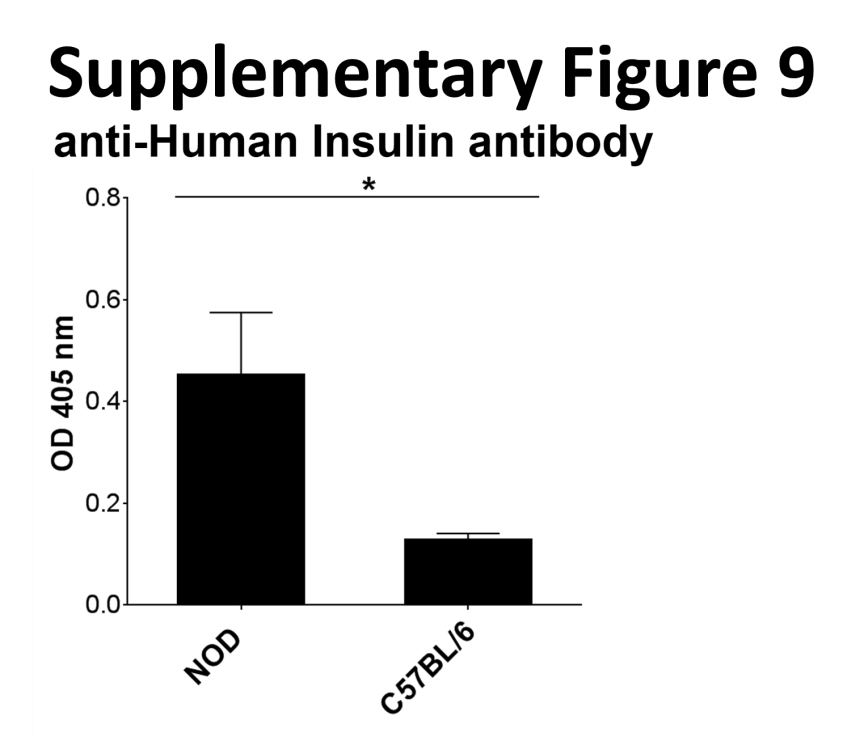


**Supplementary Fig. 9: Anti- human insulin antibody levels in healthy control NOD and C57BL/6 mice.** Mouse blood plasma tested for anti-human insulin antibodies by ELISA. The absorbances at 405 nm correlate with the concentration of the antibody. * (p≤0.05), unpaired Student’s *t* test. Data shown are expressed as mean ± SE and are representative of 2 independent experiments.


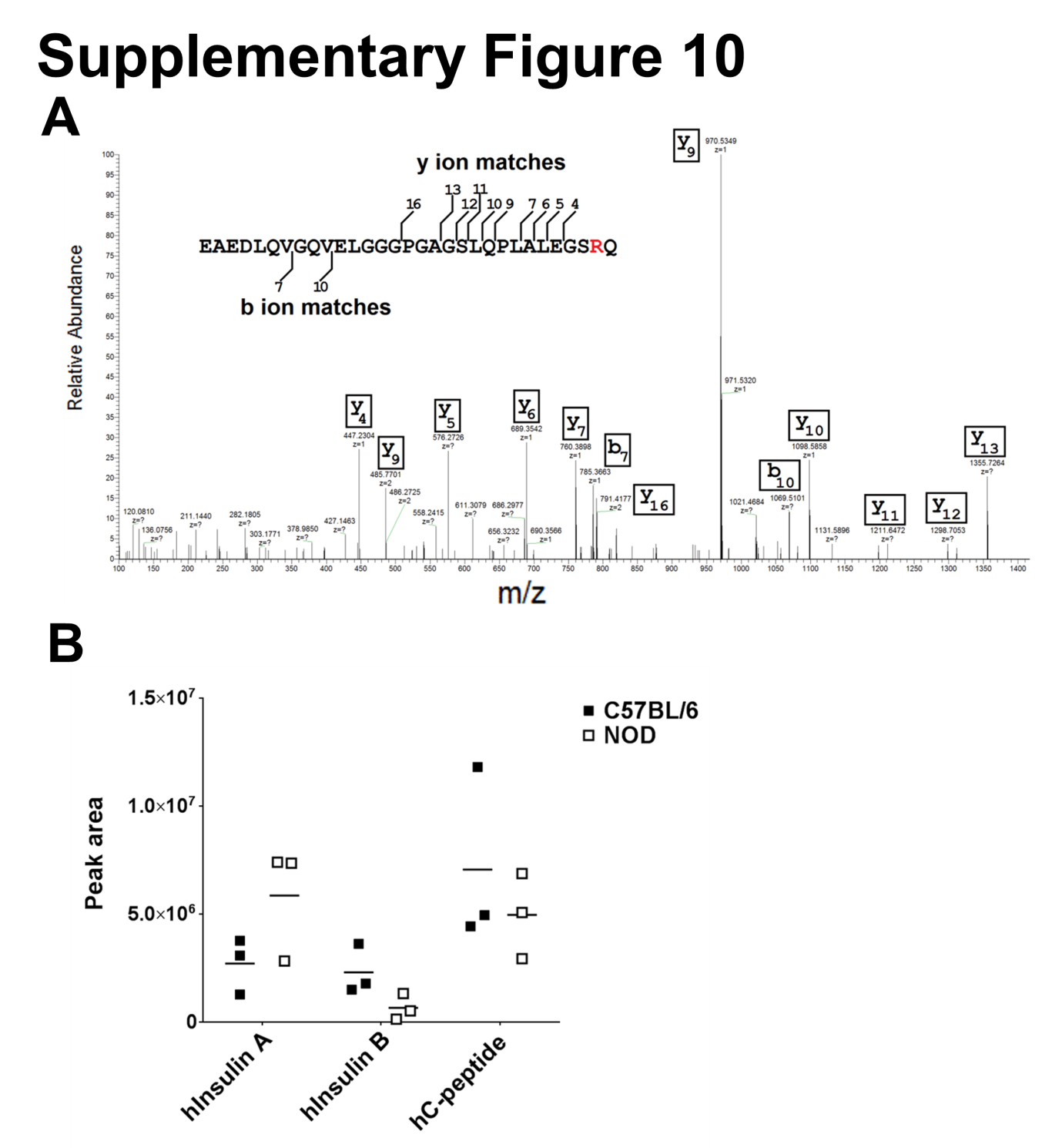


**Supplementary Fig. 10: Mass spectrometry analysis suggests that lower transgenic C-peptide production in the NOD mice does not correlate with insulin sequestration due to circulating antibodies.** (A) Product ion spectrum of the human insulin transgene C-peptide detected in the circulation of AAV2/8-HLP-hINSco treated mice. Ion *m/z* values are labelled to show matched b and y ion fragments. The red arginine residue indicates the single amino acid change from the wild type human C-peptide sequence in order to induce a furin mediated cleavage event. (B) Peak areas values for the human insulin A-, B- and C-peptides detected in the individual AAV2/8-HLP-hINSco -treated mice plasma extracts.
